# The evolutionary dynamics of haplodiploidy: Genome architecture and haploid viability

**DOI:** 10.1111/evo.12792

**Published:** 2015-11-02

**Authors:** Heath Blackmon, Nate B. Hardy, Laura Ross

**Affiliations:** ^1^ Department of Biology University of Texas Arlington, Box 19498 Arlington Texas 76019; ^2^ Department of Entomology and Plant Pathology Auburn University Auburn Alabama 36849; ^3^ School of Biological Sciences Institute of Evolutionary Biology University of Edinburgh Edinburgh EH9 3JT United Kingdom

**Keywords:** Acari, chromosomal evolution, haplodiploidy, reproductive strategies, sex determination

## Abstract

Haplodiploid reproduction, in which males are haploid and females are diploid, is widespread among animals, yet we understand little about the forces responsible for its evolution. The current theory is that haplodiploidy has evolved through genetic conflicts, as it provides a transmission advantage to mothers. Male viability is thought to be a major limiting factor; diploid individuals tend to harbor many recessive lethal mutations. This theory predicts that the evolution of haplodiploidy is more likely in male heterogametic lineages with few chromosomes, as genes on the X chromosome are often expressed in a haploid environment, and the fewer the chromosome number, the greater the proportion of the total genome that is X‐linked. We test this prediction with comparative phylogenetic analyses of mites, among which haplodiploidy has evolved repeatedly. We recover a negative correlation between chromosome number and haplodiploidy, find evidence that low chromosome number evolved prior to haplodiploidy, and that it is unlikely that diplodiploidy has reevolved from haplodiploid lineages of mites. These results are consistent with the predicted importance of haploid male viability.

Reproduction and sex determination are remarkably variable across life. Although in most species reproduction is symmetrical—mothers and fathers contribute nuclear genes equally to their offspring (barring sex chromosome‐linked loci)—this symmetry has broken down frequently. In about 15% of species, the genetic roles of mother and father during reproduction are highly imbalanced: Mothers monopolize the production of male offspring (haplodiploidy), either by the asexual production of sons (arrhenotoky*)* or by producing sons that eliminate their father's genome from their germline (paternal genome elimination, PGE). Although haplodiploidy has received substantial attention due to its tenuous role in the evolution of eusociality (Hamilton 1972; Trivers and Hare 1976; Gardner et al. 2012; Gardner and Ross 2013), its evolution remains poorly understood. This is not for lack of interest. Many authors have considered different scenarios for its evolution, and a wealth of theory has been developed on this topic (Brown 1964; Bull 1979, 1983; Borgia 1980; Sabelis and Nagelkerke 1988; Haig 1993aa,b; Goldstein 1994; Smith 2000; Normark 2004, 2006; Burt and Trivers 2006; Immler and Otto 2014). Yet few of these ideas have been tested in an empirical or comparative framework. Here, we present the first comparative analysis aimed at understanding the evolutionary dynamics of haplodiploidy, focusing both on its origin and its loss.

Most theories assume that haplodiploidy arises from maternal–paternal genetic conflict. Hartl and Brown (1970) and Bull (1979) presented the first formal models showing that when a mother is able to produce haploid sons, she benefits, as these sons always pass on her genes to their offspring (instead of only half of her genes in a diploid son). However, selection for haploid sons is counterbalanced by their expected lower viability. As a result, haplodiploidy will only spread if haploid males are at least half as viable as diploid males. This was confirmed by subsequent models, building upon this work (Bull 1983; Haig 1993aa,b; Normark 2004; Burt and Trivers 2006). Alternatively haplodiploidy could evolve in response to selection for females to be able to produce offspring when unmated or control their sex ratio (Hamilton 1967; Borgia 1980; Jordal et al. 2001). In these alternative (but not mutually exclusive) explanations, the evolution of haplodiploidy is still expected to be subject to at least some haploid male viability constraints. Most diploid individuals have a considerable number of recessive lethal mutations in their genome and therefore are unlikely to survive as a haploid (Simmons and Crow 1977; Charlesworth and Charlesworth 1999). Making the general prediction that transitions toward haplodiploidy should be very difficult indeed.

This prediction is hard to reconcile with the recurrent evolution of haplodiploidy (approximately two dozen origins) across the tree of life (Bull 1983; Otto and Jarne 2001; Normark 2003; de la Filia et al. 2015). Therefore, the question is if any genetic or ecological factors could reduce this viability effect, thereby promoting transitions toward haplodiploidy. One factor that could affect the viability of haploid males is their genome architecture. All transition from diploidy to haplodiploidy took place under male heterogamety (Bull 1983; Gardner and Ross 2014), in which males are either XO or XY. Under an XO‐male sex‐determining system (or XY with a degenerate Y), the X chromosome shows haploid gene expression. Therefore, the frequency of X‐linked recessive deleterious alleles is expected to be low. Furthermore, the overall frequency of recessive deleterious alleles (genetic load) is expected to be lower in species for which the X chromosome makes up a large proportion of the genome (White 1973; Bull 1979, 1983). Assuming that on average autosomes and X chromosomes have a similar size, this leads to the testable prediction that species with very few chromosomes (e.g., one X and two autosomes) are more likely to evolve haplodiploidy than those with many chromosomes (e.g. one X and 20 autosomes). If haploid viability is what limits transitions toward haplodiploidy, we would therefore expect a correlation between ploidy (haplodiploidy vs. diploidy) and chromosome number.

Of course the taxonomic distribution of haplodiploidy is not just dependent on factors that affect its origin, but also on those that affect its loss. There is no formal theory exploring the loss of haplodiploidy. Bull (1983) suggested that reversions back to diplodiploidy are unlikely because under haplodiploidy sperm is produced mitotically, and meiotic spermatogenesis would be hard to reevolve once lost. Indeed, based on a crude observation of the taxonomic distribution of haplodiploidy this seems to hold true, but no explicit comparative analysis has been conducted and transitions remain unclear in some taxonomic groups.

Here, we use a phylogenetic comparative framework, to address if (1) haplodiploidy evolves more readily in species with a low chromosome count and (2) if reversions back to diploidy have occured. Our analysis focuses on the Acari (mites and ticks), which are uniquely suitable for such an approach, as it is the only clade in which haplodiploidy has evolved repeatedly. We use reproductive data on 424 species of mites combined with a phylogeny of 770 species.

## Methods

### DATA COLLECTION

The Acari are considered a subclass of the Arachnida. There are about 50,000 described species, classified in two orders: the Acariformes and Parasitiformes (Dabert et al. 2010). Mite reproductive systems are diverse, ranging from diplodiploidy (with either XO or XY genetic sex determination), haplodiploidy, and parthenogenesis to a type of PGE in which males develop from fertilized eggs, but paternal chromosomes are lost during early development rendering them haploid (Norton et al. 1993; Toyoshima and Amano 2012).

We collected all published data on the reproduction, ploidy, and karyotypes of sexually reproducing mites and ticks. We focused on three important reviews of mite reproduction (Oliver 1971, 1977; Norton et al. 1993) and further supplemented this dataset with an extensive survey of the literature, by direct searches on Web of Science and Google Scholar, as well as by forward and backward citation searches. We scored the reproductive systems as a binary trait with the states haplodiploidy (either arrhenotoky or PGE) and diplodiploidy. For diploid taxa, we also noted the sex‐determination system (XY vs. XO). We also recorded the number of chromosomes (diploid chromosome count in females) for all sexually reproducing Acari species. In total we obtained reproductive data for 424 species, although the character matrix is not complete for every species. All data including the references are deposited and available on the NESCent “Tree of Sex” database (http://www.treeofsex.org, Tree of Sex Consortium 2014).

### PHYLOGENETIC RECONSTRUCTION

We downloaded phylogenetically informative DNA sequence clusters as FASTA files from PhyLoTA rel 1.5 (Sanderson 2008). We started with data from three mitochondrial genes (COI, 12S, 16S) and five nuclear genes (EF1alpha, heat‐shock protein cognate 5 [Hsc70‐5], signal recognition particle protein 54k [Srp54k], 18S, 28S) that had been sampled from 822 mite species. We used MAFFT (Katoh and Toh 2008) to align sequences from each gene. We used Gblocks (Talavera and Castresana 2007) to purge hypervariable regions from each ribosomal alignment. In each Gblocks run, we set the allowed gap position to half, the minimum block length to 5, and the maximum number of contiguous nonconserved positions to 12. We then used Mesquite version 2.73 (Maddison and Maddison 2013) to delimit codon positions and delete introns in protein‐coding alignments, as well as build a supermatrix of concatenated alignments. The total length of the supermatrix was 12,132 sites. At this stage, we removed taxa from the phylogenetic dataset that were not represented in the genetic/sexual system trait dataset. This was necessary to make phylogeny and divergence time estimation tractable. We divided the pruned supermatrix into six data partitions: first and second nuclear codon positions, third nuclear codon positions, first and second mitochondrial codon positions, third mitochondrial codon positions, nuclear ribosomal sites, and mitochondrial ribosomal sites. We used BEAST version 1.7.5 (Drummond and Rambaut 2007) to estimate phylogenetic relationships and time‐proportional branch lengths. The BEAST analysis estimated the parameters of an HKY + G nucleotide substitution model independently for each of the six data partitions. It used a birth–death model of phylogenetic branching, and an uncorrelated log‐normal relaxed clock model of among‐branch substitution rate variation (Drummond et al. 2006). We calibrated divergence times with three fossil‐based, exponential node priors: (1) a minimum age of 380 Ma on the stem node of Acariformes (Norton et al. 1988; Hirst n.d.); (2) a minimum age of 90 Ma on the stem node of Argasidae (Klompen and Grimaldi 2001); and (3) a minumum age of 35 Ma on the stem node of Ixodes (Scudder 1885). We ran the BEAST analysis for 100 million steps and sampled trees once every 10,000 steps. We examined log files in Tracer (Drummond and Rambaut 2007) and determined that MCMC sampling from the stationary distribution commenced after 70 million steps. We randomly selected 100 trees from those sampled from the stationary distribution. To account for phylogenetic uncertainty, we repeated each of the comparative phylogenetic tests over this set of 100 high posterior probability (HPP) trees.

To maximize overlap between our trait database and phylogeny for comparative approaches we used an iterative tip matching approach. Briefly, we built our trait matrix by first finding exact species matches between the taxa in our tree and database. Each genus present on our tree that had no species level matches was then collapsed to a single tip and we searched for trait data for the genus. This process was repeated at the family level as well. At all taxonomic levels, if there was more than one record in our trait database that matched a tip, we used the mean chromosome number for the tip; in each of these cases the discrete traits such as reproductive mode were the same for all matching records in the database. This approach produced 18 genus‐level matches and four family‐level matches.

### ORIGIN AND LOSS OF HAPLODIPLOIDY

A previous review of the evolution of haplodiploidy in mites suggested that it evolved repeatedly in the Acari (Norton et al. 1993). To test this hypothesis and to determine if transitions out of haplodiploidy are likely to have occurred, we reconstructed the evolution of haplodiploidy in the R package Ape (Paradis 2011). We used AIC scores to compare two models: (1) a two‐rate model allowing for different rates of transitions between haplodiploidy and diplodiploidy with stationary root frequencies and (2) a one‐rate model that only allows transitions from diplodiploidy to haplodiploidy with the root state fixed as diplodiploidy. To estimate the number of origins of haplodiploidy, we performed stochastic character mapping in the R package phytools (Revell 2012). Tests were conducted using the set of 100 HPP trees with 109 tips (the total for which both ploidy and chromosome number were available). This included 18 genus‐level matches and four family‐level matches. Tests were repeated on a second set of pruned HPP trees with only the 87 tips that were exact species‐level matches. Although both of these datasets include representatives from the Acariformes and Parasitiformes, we have more Parasitiformes, which account for approximately 63% of both datasets.

### MCMCglmm AND THRESHOLD ANALYSES

A correlation between chromosome number and ploidy could result from either faster transitions to haplodiploidy in low‐chromosome lineages, as hypothesized by Bull, or shared phylogenetic ancestry. To test for such a correlation, we analyzed the data using taxonomic and phylogenetic mixed models (Hadfield and Nakagawa 2010) in the R package MCMCglmm (Hadfield 2010). We corrected for phylogenetic nonindependence among origins by using either nested taxonomic levels (infraorder/family/genus) or the reconstructed molecular phylogeny. We used a mixed model with Gaussian error structures and log‐transformed chromosome numbers as the response variable. As predictors we included ploidy (haplodiploidy vs. diploidy). We used inverse‐gamma priors for the residual variance and parameter‐expanded priors for the random effects (Hadfield 2015b). All models were run for one million iterations with a burn‐in of 200,000 iterations. Phylogenetically corrected models were marginalized across the set of 100 HPP trees, to account for phylogenetic uncertainty. R code is provided in the Supporting Information. We report the significance of our fixed effects in terms of *P*_MCMC_, which is twice the posterior probability that the estimate is negative or positive (whichever probability is smallest). This value can be interpreted as a Bayesian equivalent to the traditional *P*‐value (Hadfield 2010; Hadfield et al. 2013).

Finally, to corroborate the results we repeated the analysis by using Felsenstein's phylogenetic threshold model (Felsenstein 2012) using the R package MCMCglmm RAM (Hadfield 2015aa). The threshold model is similar to the phylogenetic mixed model described above, but differs in that it assumes that the phylogenetic heritabilities (Pagel's λ) of both traits are 1, instead of estimating them from the data. For the threshold model, we present the estimated correlation between chromosome number and ploidy as well as the 95% credibility intervals.

### DOES CHROMOSOME NUMBER AFFECT PLOIDY EVOLUTION? OR, DOES PLOIDY AFFECT THE EVOLUTION OF CHROMOSOME NUMBER?

The MCMCglmm and threshold models described above allow us to estimate the correlation between chromosome number and ploidy. However, they do not allow us to test the directionality of the causation, that is, determine which evolves first: low chromosome number or haplodiploidy. Such analyses are notoriously difficult in a phylogenetic context (Maddison and FitzJohn 2015), especially when the hypothesis is that a continuous trait affects the evolution of a discrete trait. Therefore, we present two separate approaches, each with their own advantages and drawbacks. (1) We used a simple taxonomic approach (as described in Ross et al. 2012) in which we compare the average chromosome number between infraorders that have or do not have haplodiploid taxa nested within them. If haplodiploidy evolves as a consequence of low chromosome counts, we would expect diploid taxa in infraorders harboring haplodiploids to have fewer chromosomes than diploids in infraorders without haplodiploids. (2) We used a phylogeny‐based version of the taxonomic approach, that is, a method that assesses if the mean chromosome number at nodes subtending the origin of haplodiploidy is lower than expected if the traits were evolving independently. In specific, we used the R package phytools (Revell 2012) to reconstruct the evolution of haplodiploidy using stochastic character mapping. Then, we reconstructed ancestral ploidy over a set of 100 HPP trees. Next, we pruned the haplodiploid taxa from each of the HPP trees, and used the R package Ape (Paradis 2011) to conduct a maximum‐likelihood reconstruction of the evolution of chromosome number on each of the pruned trees, assuming that chromosome number evolves under Brownian Motion. We used a Monte Carlo approach with 100 iterations on each ancestral state reconstruction to produce a null distribution for the mean ancestral chromosome number at randomly chosen nodes. We then compared the mean number of chromosomes at the nodes that led to haplodiploid clades to this null distribution.

## Results

### ORIGINS AND LOSS OF HAPLODIPLOIDY WITHIN THE ACARI

**Figure 1:**
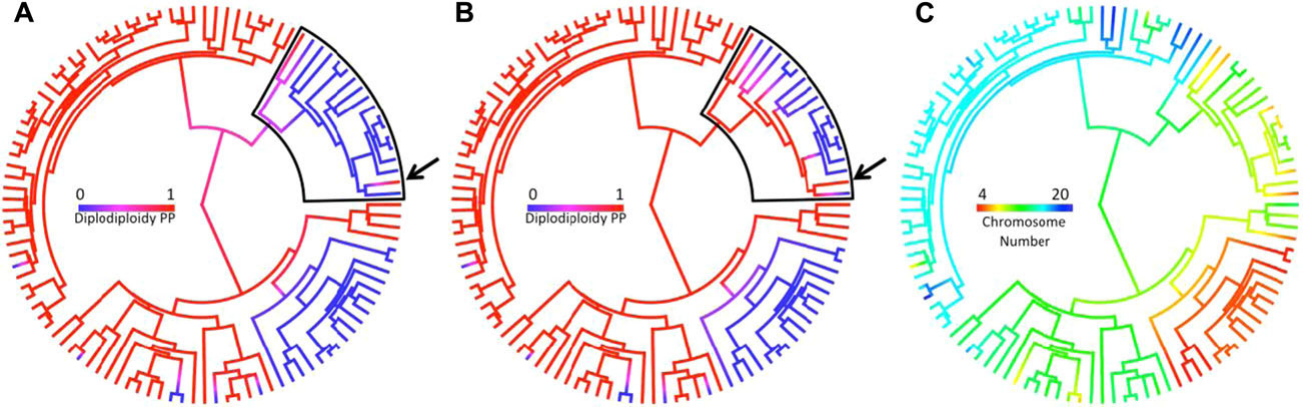
Ancestral state reconstructions. (A) Posterior probability of diplodiploidy under a model in which transition from haplodiploidy (blue) to diploidy (red) is allowed. (B) Posterior probability of diplodiploidy under a model in which transition from haplodiploidy to diplodiploidy is not possible. Black boxes in (A) and (B) indicate the portion of the tree in which reconstructions differ between models. (C) Maximum‐likelihood reconstruction of diploid chromosome number. The arrow in (A) and (B) indicates the family Parasitidae that was removed for one analysis.

Our analysis shows that haplodiploidy has evolved multiple times in Acari. Ancestral state reconstruction under a one‐rate model in which only transitions from diplodiploidy to haplodiploidy are allowed suggests a mean of 12.9 origins. Using a two‐rate model in which reversions from haplodiploidy to diplodiploidy are also allowed, we infer a mean of 7.9 origins of haplodiploidy. However, we find only limited support for the hypothesis that transitions from haplodiploidy to diplodiploidy are possible. Using the HPP tree set with all 109 taxa included, we calculate a mean AICc difference of –8.04 indicating that the two‐rate model allowing for transition from haplodiploidy to diplodiploidy is the best fit for the data. However, when we prune these trees to keep only the 87 exact species‐level matches, the mean AIC difference is 4.21, indicating that the model in which transition from haplodiploidy to diplodiploidy is not possible is the best fit. One of the family‐level matches (Parasitidae sp.) on our HPP trees is reported as diplodiploidy but is deeply nested among haplodiploidy taxa. To test the degree to which this single tip was driving the difference in our results, we repeated our analysis removing only this tip from HPP tree set. Model comparison across these trees produced a mean AICc difference of 4.06, indicating that this single tip is producing all support for reversibility of haplodiploidy. Figure 1 shows the ancestral state reconstruction of haplodiploidy under the two‐rate model in which reversions are possible (Fig. 1A) and the one‐rate model in which they are not (Fig. 1B).

### HAPLODIPLOIDY AND CHROMOSOME NUMBER

**Figure 2:**
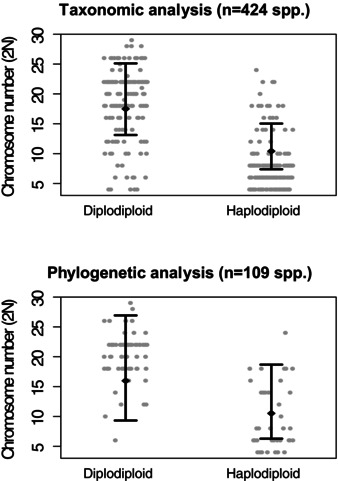
The relationship between chromosome number and ploidy across the Acari. (A) Scatterplot of all available estimates of chromosome number of haplodiploid and diplodiploid taxa for which taxonomic data were available. The black diamonds and error bars show the model prediction (posterior mean and 95% CI) of a taxonomic mixed model in MCMCglmm. (B) Scatterplot of all available estimates of chromosome number of haplodiploid and diplodiploid taxa for which phylogenetic data were available. The black diamonds and error bars show the model prediction (posterior mean and 95% CI) of a phylogenetic mixed model in MCMCglmm.

We compared the diploid chromosome count of diploid and haplodiploid species using a taxonomic mixed modeling approach (424 sp.) and a phylogenetic mixed model (109 taxa, 87 species‐level matches, 18 genus‐level matches, and four family‐level matches). Both models confirm that haplodiploid species have significantly lower chromosome numbers than diploid species (approximately 2*n* = 5 fewer chromosomes, *P*_MCMC_ < 0.001 for both the taxonomic and phylogenetic models, see Fig. 2A, B). We also estimated the phylogenetic heritability (akin to Pagel's λ) of chromosome number as 0.91 (95% CI: 0.79–0.96). Using a threshold model (assuming λ = 1 for both chromosome number and ploidy), we confirmed these results and recovered a strong negative correlation between chromosome number and ploidy (–0.83, 95% CI: –0.91 to –0.53). Next we attempt to determine what came first: low chromosome number or haplodiploidy. Determining the direction of causality in a phylogenetic framework is difficult; therefore we here present the results of two alternative approaches.

First of all we simply compare the average chromosome number of diploids between infraorders that have or do not have haplodiploid taxa nested within them. We are able to estimate this parameter for seven infraorders, four of which include haplodiploids (see Supporting Information). As we would expect, if low chromosome number increases the probability of transitioning toward haplodiploidy, we find that diploids within infraorders that include haplodiploid taxa on average have a lower chromosome numbers (2*n* = 15) than those within infraorders in which haplodiploid did not originate (2*n* = 19). However, this difference is not significant (*t*_(4.26)_ = 1.94, *P* = 0.119), perhaps because of the limited statistical power of this analysis.

We therefore also address this problem with a more powerful phylogenetic approach: Using a combination of maximum‐likelihood ancestral state reconstructions of chromosome number (Fig. 1C) and stochastic mappings of sexual system, we tested whether haplodiploid clades originate from nodes with low numbers of chromosomes. We performed this test on both our full dataset with 109 taxa and our dataset with 87 exact species‐level matches. Using the full dataset stochastic mappings infer an average of 10 origins of haplodiploidy, which arose with a mean diploid chromosome number of 18.4. If chromosome number and ploidy evolved independently we would expect a mean of 20.2 (*P* = 0.017). Next we performed our test using only data from the 87 exact species‐level matches. Using this dataset, we infer an average four origins of haplodiploidy with a mean chromosome number of 18.6. If chromosome number and ploidy evolved independently we would expect a mean of 20.7 (*P* = 0.038). These results are consistent with Bull's (1983) hypothesis that haplodiploidy does arise more frequently in clades with fewer chromosomes.

## Discussion

In this study, we address the evolutionary dynamics of haplodiploidy in mites using a comparative approach. We show that haplodiploidy has evolved repeatedly in mites and take advantage of this evolutionary replication to study the directional bias of these transitions, as well as the correlates that might explain them. Explanations for the evolution of haplodiploidy commonly assume it evolved because the production of haploid males provides a transmission advantage to mothers (Brown 1964; Hartl and Brown 1970; Bull 1979). This selection pressure, in principle should apply to all sexually reproducing organisms, raising the question why haplodiploidy is not universal (Gardner and Ross 2014). The answer most likely is that the transition is accompanied by strong viability costs: haploid males express recessive deleterious mutations previously masked in diploids (Bull 1983; Otto and Jarne 2001).

Here, we considered how genome architecture might cause some taxa to suffer a lower viability cost than others. Haplodiploidy tends to evolve from male heterogamety (Bachtrog et al. 2014) and as a result X‐linked deleterious recessive mutations are purged in males (Vicoso and Charlesworth 2006). Therefore, species with a low chromosome count—in which a large proportion of genes are X‐linked—might suffer a lower viability cost of haploid males (Bull 1983). To test this hypothesis, we estimated the correlation between chromosome number and the presence of haplodiploidy in mites in which the haploid chromosome count varies from *n* = 2 to *n* = 14 and found a strong correlation: Haploid taxa have on average about one‐third fewer chromosomes than their diploid counterparts (Fig. 2). Thus, the phylogenetic distribution of haplodiploidy among mites matches the theoretical predictions (Bull 1983) that species with lower chromosome counts will be more likely to make the transition to haplodiploidy.

However, there are alternative explanations for this correlation. First of all, it is possible that the difference in karyotype arose as a result of, and not prior to, the evolution of haplodiploidy. We have used two different analyses to distinguish between these two scenarios. Both of these suggest that low chromosome counts most likely preceded the evolution of haplodiploidy. Unfortunately, phylogenies provide very little information about the timing of coevolutionary events, so the statistical support is relatively weak. There are, however, no clear predictions on why haplodiploidy would select for a reduction in chromosome number. In fact, theory might suggest the opposite (Wilfert et al. 2007): Under haplodiploidy there is no recombination in males, so if recombination rate is under stabilizing selection, then haplodiploidy might select for an increased recombination rate in females. There is indeed limited evidence of higher recombination rates among haplodiploids (Wilfert et al. 2007). Chromosome number is positively correlated with recombination rate (Lynch 2008), suggesting selection for an increase and not a decrease in chromosome number under haplodiploidy (Ross et al. 2015).

If a decline in chromosome number often precedes the evolution of haplodiploidy, what factors promote this reduction in the first place? Reductions in chromosome number are probably the result of fusions between chromosomes. Unfortunately, the adaptive advantages of chromosomal fusions are unclear. One hypothesis is that low chromosome numbers allow the maintenance of coadapted gene complexes, since it minimizes recombination (Stebbins 1971). An alternative explanation, applicable to groups in which there is meiotic drive, is that biased transmission dynamics can promote the aggregation of genetic material onto a driver element, and this process could result in the fusion of entire chromosomes (Pardo‐Manuel and Sapienza 2001). Unfortunately, we have no way of testing these hypotheses without more information about mite genomes.

Although chromosome number is lower in all haplodiploid clades compared to their sister group, the effect is more pronounced among those within the order Acariformes than in those within the Parasitiformes (Fig. S1). One important assumption for our analysis is that the X chromosome on average is similar in size to autosomes. However, in male heterogametic species of Parasitiformes, the X chromosome is often several times larger than the autosomes, whereas among Acariformes, the X chromosome tends to be smaller than the autosomes (Oliver 1977). So, even with same numbers of chromosomes, a larger proportion of genes might be X‐linked in Parasitiformes than in Acariformes.

Haplodiploidy is often thought to evolve through genomic conflicts between parents (Bull 1983; Gardner and Ross 2014). However, mutation clearance might further aid transition toward haplodiploidy (Goldstein 1994). Haploid males express recessive mutations, allowing more efficient selection to purge deleterious and fix beneficial mutations. This could lead to the buildup of linkage between the locus that causes haplodiploidy and beneficial alleles at other loci—an effect particularly pronounced under low recombination rates. Therefore, if we assume again that chromosome number correlates with recombination rate, this process predicts that low chromosome counts might further promote the evolution and maintenance of haplodiploidy.

Of course the phylogenetic distribution of haplodiploidy is not just determined by the transition rate from diploidy to haplodiploidy, but also by possible reverse transitions. Transitions from haplodiploidy back to diploidy are generally thought to be rare, due to the difficulty associated with reevolving meiotic spermatogenesis (Bull 1979, 1983). Indeed this transition has never been observed among insect or nematodes (Normark 2003), but does it occur among mites? We used an ancestral state reconstruction approach to address this and found no strong support for transitions from haplodiploidy to diploidy, although the quality of our data did not allow us to rule out this possibility completely. Upon further investigation, it seems that the only potential reversal (the diploid family Parasitidae) we identified is nested with a clade with PGE rather than arrhenotoky (Fig. S2). Reversal from PGE is thought to be less evolutionarily constrainted and known to occur among several scale insects (Hemiptera: Coccoidea, Ross et al. 2010).

Several authors previously considered the evolution of haplodiploidy in mites. An important focus of these earlier studies was the evolutionary relationship between PGE and arrhenotoky. Cruickshank and Thomas (1999) used a comparative analysis to show that, within the Dermanyssina mites, arrhenotoky evolved from PGE rather than directly from diploidy. However, this result is not strongly supported in our data (see Fig. S2). Furthermore there is no reliable evidence for PGE outside the Dermanyssina (Norton et al. 1993), so a possible transition from PGE to arrhenotoky could only explain one of the eight to 12 origins we infer. In his discussion of haplodiploidy in mites, Norton et al. (1993) postulates a number of alternative factors that could promote the evolution of haplodiploidy in mites, most importantly mating between relatives. Sib mating is common in some, but not all haplodiploid clades (Borgia 1980; Gardner and Ross 2014). Inbreeding and resulting selection for female‐biased sex ratio is thought to play a role in the evolution of PGE (Gardner and Ross 2014), but its role in the evolution of arrhenotoky is less straightforward. Inbreeding purges recessive deleterious alleles, thereby potentially increasing haploid male viability and reducing the importance of chromosome number. Therefore, taking into account patterns of inbreeding as well as chromosome number might allow us to better predict the distribution of haplodiploidy among mites. However, inbreeding could also evolve as a result of haplodiploidy: Arrhenotokous species might be more likely to evolve mating strategies involving inbreeding, as they are less likely to suffer from inbreeding depression (Norton et al. 1993).

Here we have shown that sex‐determination system and genome architecture can affect the evolution of haplodiploidy. In doing so we were able to predict, at least in part, the phylogenetic distribution of ploidy within the mites. It is unclear if chromosome number can also predict its phylogenetic distribution in other clades. It is tantalizing that extremely low chromosome numbers (*n* < 3) seem to commonly occur among haplodiploid clades (Oliver and Nelson 1967; Crosland and Crozier 1986; Tree of Sex Consortium 2014). On the other hand, the combination of male‐heterogametic sex determination and low chromosome numbers occurs across large clades of invertebrates that do not appear to have evolved haplodiploidy. This suggests that these factors alone are insufficient to explain broader phylogenetic patterns of haplodiploidy. Further, theoretical and comparative work will be necessary to determine additional evolutionary correlates of haplodiploidy, as to fully unravel its evolution.

## Supporting information


**Figure S1**. An exemplar tree from our HPP tree set.
**Figure S2**. Maximum‐likelihood ancestral state reconstruction of sexual system in the clade Dermanyssiae.

**Table S1**. Summary tables of MCMCglmm analyses.
**Table S2**. Determining the direction of causality between ploidy and chromosome number.
