# Supplementary figures and images for: The evolutionary dynamics of haplodiploidy: Genome architecture and haploid viability
Source: Evolution. 2015 Nov 2;69(11):2971–8. doi: 10.1111/evo.12792 (PMC4989469; doi:10.1111/evo.12792)

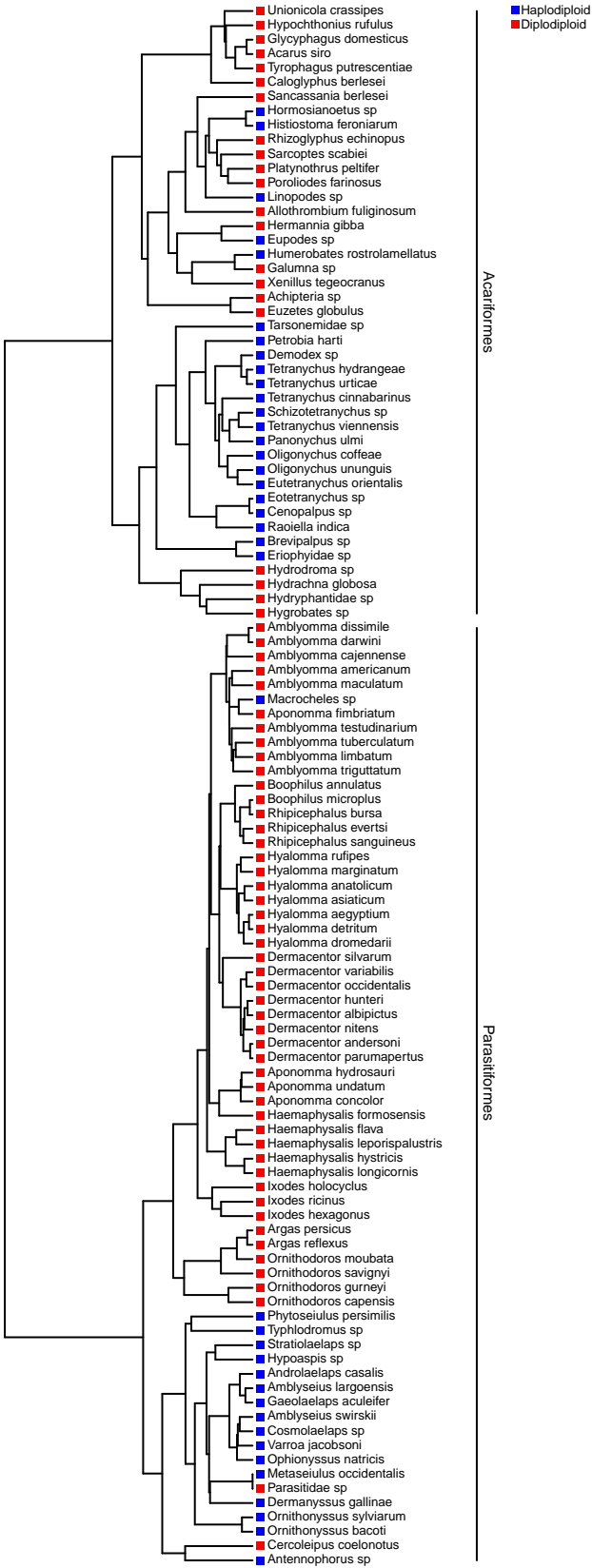

Supplement: Supplementary file 1 — Figure S1. An exemplar tree from our HPP tree set. Figure S2. Maximum‐likelihood ancestral state reconstruction of sexual system in the clade Dermanyssiae. [file EVO-69-2971-s001.pdf]

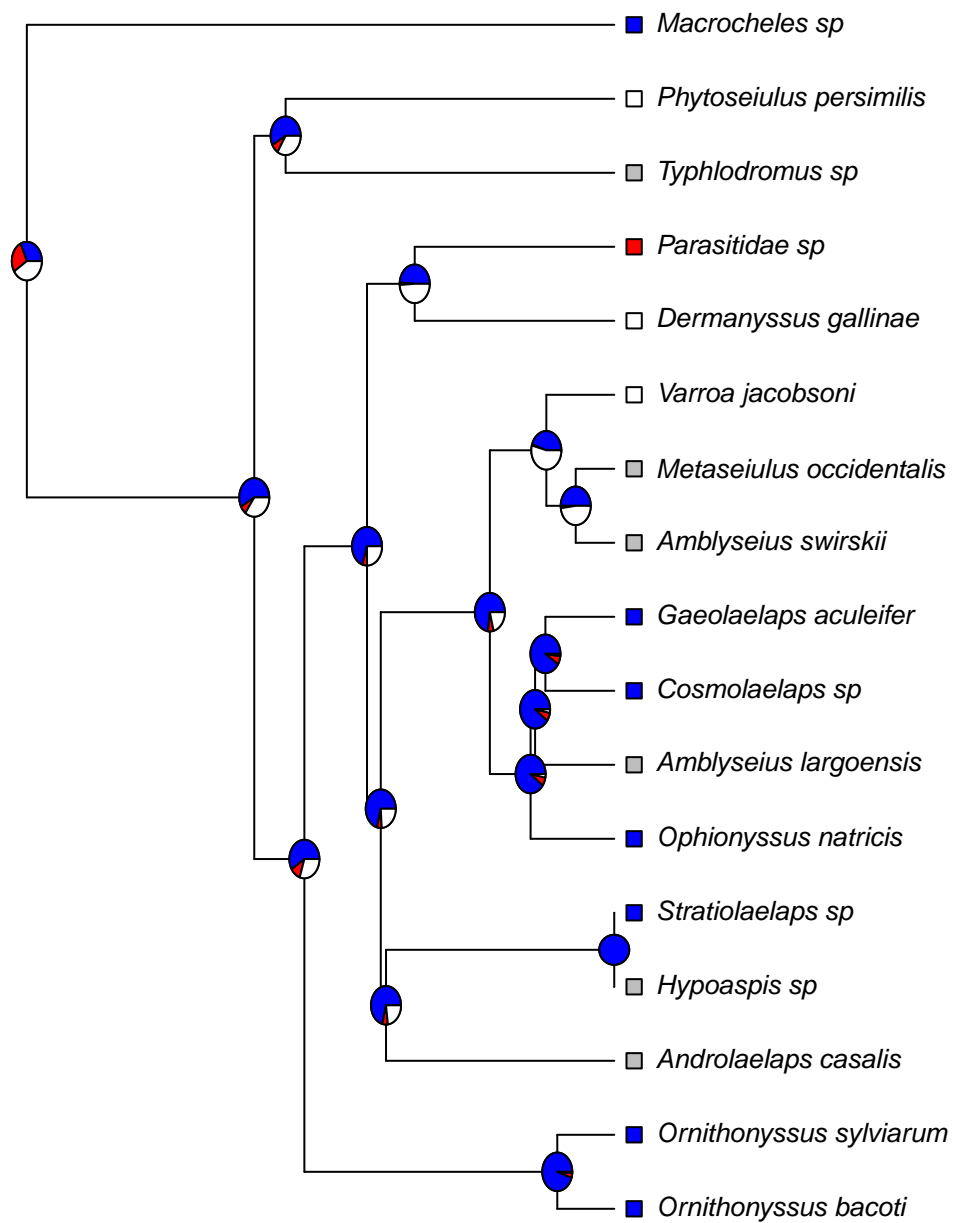

- Paternal genome elimination
- Haplodiploidy – unspecified type
- Arrhenotoky
- Diplodiploidy

Supplement: Supplementary file 2 — Table S1. Summary tables of MCMCglmm analyses. Table S2. Determining the direction of causality between ploidy and chromosome number. [file EVO-69-2971-s002.pdf]
